# Up-regulated expression of *l*-caldesmon associated with malignancy of colorectal cancer

**DOI:** 10.1186/1471-2407-12-601

**Published:** 2012-12-17

**Authors:** Kyung-Hee Kim, Seung-Gu Yeo, Won Ki Kim, Dae Yong Kim, Hyun Yang Yeo, Jun Pyu Hong, Hee Jin Chang, Ji Won Park, Sun Young Kim, Byung Chang Kim, Byong Chul Yoo

**Affiliations:** 1 Colorectal Cancer Branch, Division of Translational and Clinical Research I, Research Institute, National Cancer Center, Goyang, 410-769, Republic of Korea; 2 Laboratory of Cell Biology, Cancer Research Institute, Seoul National University College of Medicine, Seoul, 110-744, Republic of Korea; 3 Department of Radiation Oncology, Soonchunhyang University College of Medicine, Cheonan, 330-721, Republic of Korea; 4 Center for Colorectal Cancer, Hospital, National Cancer Center, Goyang, 410-769, Republic of Korea

## Abstract

**Background:**

Caldesmon (CaD), a major actin-associated protein, is found in smooth muscle and non-muscle cells. Smooth muscle caldesmon, *h*-CaD, is a multifunctional protein, and non-muscle cell caldesmon, *l*-CaD, plays a role in cytoskeletal architecture and dynamics. *h*-CaD is thought to be an useful marker for smooth muscle tumors, but the role(s) of *l*-CaD has not been examined in tumors.

**Methods:**

Primary colon cancer and liver metastasis tissues were obtained from colon cancer patients. Prior to chemoradiotherapy (CRT), normal and cancerous tissues were obtained from rectal cancer patients. Whole-tissue protein extracts were analyzed by 2-DE-based proteomics. Expression and phosphorylation level of main cellular signaling proteins were determined by western blot analysis. Cell proliferation after CaD siRNA transfection was monitored by MTT assay.

**Results:**

The expression level of *l*-CaD was significantly increased in primary colon cancer and liver metastasis tissues compared to the level in the corresponding normal tissues. In cancerous tissues obtained from the patients showing poor response to CRT (Dworak grade 4), the expression of *l*-CaD was increased compared to that of good response group (Dworak grade 1). In line with, *l*-CaD positive human colon cancer cell lines were more resistant to 5-fluorouracil (5-FU) and radiation treatment compared to *l*-CaD negative cell lines. Artificial suppression of *l*-CaD increased susceptibility of colon cancer cells to 5-FU, and caused an increase of p21 and c-PARP, and a decrease of NF-kB and p-mTOR expression.

**Conclusion:**

Up-regulated expression of *l*-CaD may have a role for increasing metastatic property and decreasing CRT susceptibility in colorectal cancer cells.

## Background

Caldesmon (CaD), a major actin-associated protein, is found in smooth muscle cells (*h*-CaD; high molecular weight, 89–93 kDa) and non-muscle cells (*l*-CaD; low molecular weight, 59–63 kDa) [1,2]. At least two *h*-CaD and four *l*-CaD isoforms are produced by alternative splicing [3]. Smooth muscle caldesmon, *h*-CaD, is a multifunctional protein which binds tightly and specifically to actin, calmodulin, tropomyosin, and myosin [4-6]. It is also a substrate for many protein kinases and is thought to regulate cellular contraction [7].

The expression of *h*-CaD is specific for smooth muscle cells and soft tissue smooth muscle tumors, and in contrast to other muscle markers, it is not expressed in myofibroblasts or pericytes [8]. It was reported that *h*-CaD is present only in smooth muscle tumors, among various soft tissue tumors [9]. Thus, *h*-CaD is thought to be an extremely useful marker for smooth muscle tumors and has been used to identify soft tissue tumors with myofibroblastic characteristics [10].

The cytoskeletal structure of endothelial cells regulates their adhesive interactions with neighboring cells and the extracellular matrix. These interactions in turn control endothelial permeability and vessel wall integrity [11,12]. In non-muscle cells, *l*-CaD plays a role in cytoskeletal architecture and dynamics [13]. Although most experimental data have been obtained using *h*-CaD, the properties of *l*-CaD are expected to be quite similar [2,14].

Splice variants of *l*-CaD are differentiated by the inclusion (Hela *l*-CaD) or exclusion (WI-38 *l*-CaD) of exon 1. The results of a cohort study of cancers derived from various organs, including colon and stomach, suggested that Hela *l*-CaD could be used as a marker for angiogenic endothelial cells during the early stages of tumor neovascularization [15].

In a recent proteome assessment we found the clue that aberrant expression of CaD isoforms in colon cancer may link to tumor malignancy. We herein report altered expression of CaD in tissues from the patients with colon cancer, and discuss its possible effects on tumor malignancy, such as poor response to chemoradiation therapy.

## Methods

### Tissues from colorectal cancer patients

Fresh tissues (normal colon mucosa, primary colon tumor, normal liver, and metastatic liver tumor) were obtained from colon cancer patients who had synchronous liver metastasis. After necrotic exudates and stromal components were dissected, the overall cellularity of the normal epithelium and tumors was more than 75%. Fresh tissues (normal and cancerous) from rectal cancer patients were also obtained prior to preoperative chemoradiotherapy. Tumor regression grade was classified histologically according to the regression system of Dworak [16] as follows: grade 0, no regression; grade 1, minor regression (dominant tumor mass with obvious fibrosis in ≤ 25% of the tumor mass); grade 2, moderate regression (dominant tumor mass with obvious fibrosis in 26–50% of the tumor mass); grade 3, good regression (dominant fibrosis outgrowing the tumor mass, i.e., > 50% tumor regression); and grade 4, total regression (no viable tumor cells, only fibrotic mass). Patient characteristics are presented in Table 1. This study was approved and conducted in accordance with the guidelines by the Institutional Review Board of National Cancer Center, Korea.

**Table 1:** Characteristics of the study participants

**Sample no.**	**Sex**	**Age**	**T**	**N**	**M**	**CEA (ng/mL)**	**Grade**	**Location**
CP1	F	55	4	2	1	4.3	Low	S-colon
CP2	M	65	4	2	1	18.5	Low	Rectum
CP3	F	34	3	0	0	0.7	Low	S-colon
CP4	M	55	2	0	0	1.5	Low	Rectum
CP5	F	66	3	1	1	33.7	Low	S-colon
CP6	M	66	3	1	0	3.9	Low	S-colon
CP7	F	66	3	1	1	33.7	Low	S-colon
CP8	F	52	3	1	1	3.2	Low	A-colon
CP9	M	51	4	2	1	14.9	Low	S-colon
CP10	M	64	4	2	1	60.2	Low	A-colon
CP11	F	64	3	0	1	50.7	Low	Rectum
CP12	M	53	4	1	1	3.3	Low	S-colon
CP13	F	46	4	1	1	9.3	Low	A-colon
CP14	M	52	3	2	1	4.5	Low	Rectum
CP15	F	58	3	2	1	145.4	Low	S-colon
CP16	M	73	4	1	1	129.0	Low	Rectum
RP17	F	65	3	1	0	26.9	Low	Rectum
RP18	M	51	3	2	0	3.5	Low	Rectum
RP19	M	53	3	1	0	0.8	Low	Rectum
RP20	M	52	3	0	0	1.7	Low	Rectum
RP25	F	70	4	2	0	5.4	Low	Rectum
RP26	M	45	3	0	0	2.7	Low	Rectum
RP27	M	70	3	2	0	10	Low	Rectum
RP28	M	49	3	2	0	3.7	Low	Rectum
RP33	M	59	3	1	0	12.5	Low	Rectum
RP34	M	63	3	1	0	5.7	Low	Rectum
RP35	F	38	4	1	0	1.9	Low	Rectum
RP21	M	51	0	0	0	1.6	Low	Rectum
RP22	M	56	0	0	0	4.3	Low	Rectum
RP23	M	64	0	0	0	6	Low	Rectum
RP24	M	72	0	0	0	3	Low	Rectum
RP29	M	75	0	0	0	2.2	Low	Rectum
RP30	F	42	0	0	0	1	Low	Rectum
RP31	M	42	0	0	0	1.3	Low	Rectum
RP32	M	55	0	0	0	2.6	Low	Rectum
RP36	F	52	0	0	0	2.2	Low	Rectum
RP37	F	63	0	0	0	2.5	Low	Rectum
RP38	F	57	0	0	0	2.9	Low	Rectum

### Two-dimensional gel electrophoresis, matrix-assisted laser desorption/ionization mass spectrometry, and database searching

Two-dimensional gel electrophoresis (2-DE) was performed as described previously [17]. Briefly, samples (150 μg) of proteins extracted from colon mucosa and colon tumor tissues were applied to 13-cm immobilized pH 3–10 non-linear gradient strips (Amersham, Uppsala, Sweden) and focused at 8,000 V for 3 h. Second-dimension separation was performed in 12% polyacrylamide gels (chemicals from Serva, Heidelberg, Germany and Bio-Rad, Hercules, CA). The 2-DE gels were stained with Colloidal Coomassie Blue (Invitrogen, Carlsbad, CA) for 24 h and then destained with deionized water. Images of the 2-DE gels were analyzed using Melanie 4 software (Swiss Institute of Bioinformatics, Geneva, Switzerland). The 2-DE protein spots that showed differential expression were subjected to matrix-assisted laser desorption/ionization mass spectrometry (MALDI-MS), performed as described previously [17]. Briefly, gel pieces containing proteins of interest were excised, destained with 50% acetonitrile in 0.1 M ammonium bicarbonate, and dried in a SpeedVac evaporator. Dried gel pieces were rehydrated by incubation in 30 μl of 25 mM sodium bicarbonate, pH 8.8, containing 50 ng of trypsin (Promega, Madison, WI), overnight at 37°C. α-Cyano-4-hydroxycinnamic acid (20 mg) (Bruker Daltonics, Bremen, Germany) was dissolved in 1 ml of acetone:ethanol (1:2, v/v), and 0.5 μl of the matrix solution was mixed with an equivalent volume of sample. MALDI-MS was performed using an Ultraflex TOF/TOF system (Bruker Daltonics) operated in the positive ion reflection mode. Each spectrum was the cumulative average of 250–450 laser shots. Mass spectra were initially calibrated in closed external mode using Peptide Calibration Standard II (Bruker Daltonics) and sometimes in internal statistical mode to achieve maximum calibration mass accuracy. The spectra were analyzed using FlexAnalysis software, version 2.4 (Bruker Daltonics). Peptide mass peaks from each spectrum were submitted for a Mascot peptide mass fingerprinting search (http://www.matrixscience.com) and analyzed using BioTools software version 3.0 (Bruker Daltonics). Searches included peaks with a signal-to-noise ratio > 3. To identify proteins, the peak list for each sample was used to query the non-redundant Mass Spectrometry Protein Sequence Database. Standard settings included: enzyme (trypsin), missed cleavage (one), fixed modifications (none selected), variable modifications (oxidized methionine), protein mass (blank), mass values (MH+, monoisotopic), and mass tolerance (variable, 75 and 100 ppm).

### Western blot analysis

Western blot analysis was performed using a standard protocol [17]. Briefly, cell homogenates containing equivalent amounts of protein were centrifuged at 4,000 × g, and the supernatant fractions were subjected to SDS-PAGE. The separated proteins were transferred to polyvinylidene fluoride membranes (Millipore, Billerica, MA), which were blocked by incubation for 2 h at 4°C in 1% Tween 20-TBS buffer containing 1.5% non-fat dry milk (Bio-Rad) and 1 mM MgCl_2_. Next, the membranes were incubated with primary antibodies against caldesmon (detecting both *h*-CaD and *l*-CaD) (Abcam, Cambridge, UK), c-caspase-9 (Cell Signaling Technology, Danvers, MA), c-PARP (Cell Signaling Technology), p53 (Cell Signaling Technology), p21 (Cell Signaling Technology), NF-κB (Cell Signaling Technology), p-mTOR (Cell Signaling Technology), p-ERK (Cell Signaling Technology), p-PI3K (Cell Signaling Technology), p-AKT (Cell Signaling Technology) or β-actin (Abcam) for 2 h at room temperature, washed for 3 × 15 min with blocking solution, and then incubated with diluted horseradish peroxidase-conjugated secondary antibody (Southern Biotech, Birmingham, UK) for 1 h at room temperature. After being washed with blocking solution (3 × 15 min), the membranes were incubated with WEST-ZOL® plus chemiluminescence reagent (iNtRON Biotechnology, Gyeonggi, Korea) for 1 min and exposed to film (Kodak Blue XB-1).

### Human colon cancer cell lines

Fourteen human colon cancer cell lines (SNU-81, SNU-407, SNU-769A, SNU-769B, SNU-C4, SNU-C5, CaCo2, DLD-1, HCT116, LoVo, NCI-H508, NCI-H747, SW480 and SW620) were obtained from the Korean Cell Line Bank (Seoul, Korea) [18,19].

### MTT assay

A colorimetric assay using the tetrazolium salt, MTT, was used to assess cell proliferation after treatment with 5-FU or radiation. Equivalent numbers of cells (5 × 10^3^ cells/well) were incubated in 0.2 ml culture medium in each well. After 1, 2, 3 or 4 days of culture, 0.1 mg MTT was added to each well, followed by incubation at 37°C for a further 4 hr. Plates were centrifuged at 450 × g for 5 min at room temperature and the medium removed. Dimethyl sulfoxide (0.15 ml) was added to each well to solubilize crystals, and plates immediately read at 540 nm using a scanning multiwell spectrometer (Bio-Tek instruments Inc. Winooski, VT). Proliferation rate was obtained from six biological replicates, and all experiments were performed three times.

### *In vitro* invasion assay

An *in vitro* invasion assay was performed using a kit (Chemicon, Temecula, CA), according to the manufacturer’s protocol. Invasiveness was evaluated by staining cells that had migrated through the extracellular matrix layer and adhered to the polycarbonate membrane at the bottom of the insert. Numbers of cells adhering to six different regions of the bottom of the insert were counted at 200 × magnification.

### Small interfering RNA synthesis and transfection

The target sequences used to generate siRNA (Qiagen, Chatsworth, CA) were 5^′^-CTGAGGGAGCCTCCCAAATAA-3^′^ for *l*-CaD and 5^′^-AATTCTCCGAACGTGTCACGT-3^′^ for the non-silencing control. Transfection of siRNA was performed using HiferFect transfection reagent (Qiagen, Hilden, Germany), according to the manufacturer’s instructions. Briefly, 2 μl of 20 μM siRNA solution and 20 μl transfection reagent were incubated in 100 μl of serum-free RPMI 1640 medium for 10 min to facilitate complex formation. The resulting mixture (final concentration, 5 nM) was added to SNU-C4, a human colon cancer cell line (4 × 10^5^), and incubated in a 60 mm tissue culture dish with 4 ml of RPMI 1640.

### Statistical analysis

Between-group differences were analyzed using the non-parametric Mann–Whitney *U* test, and within-group correlations were calculated using the Spearman rank coefficient. Significance was set at *P* < 0.05.

## Results

### Differential expression of caldesmon between normal colon mucosa samples and colon tumors obtained from colorectal cancer patients

**Figure 1:**
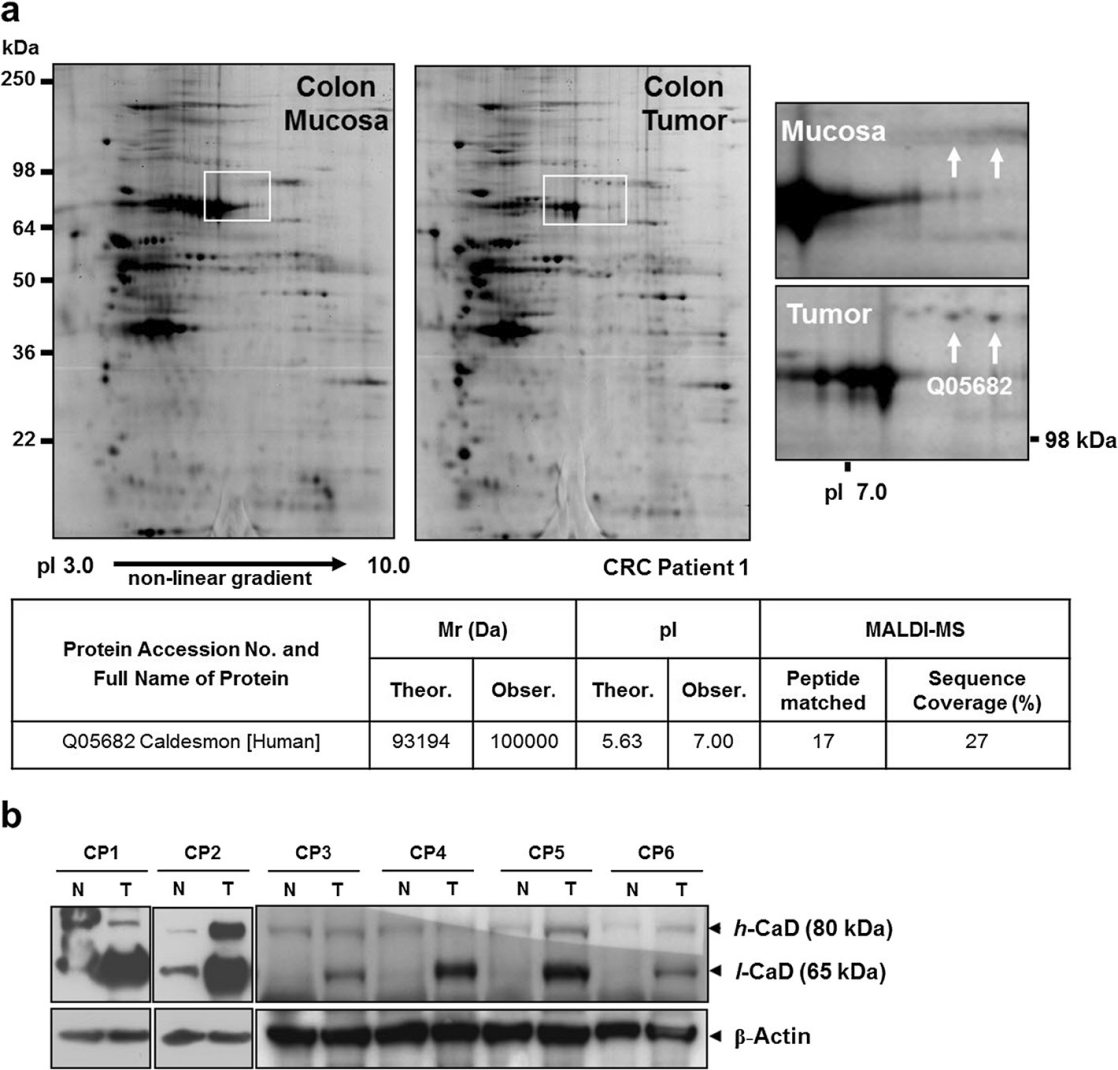
**A 2-DE-based comparative proteome analysis of normal colon mucosa samples and colon tumors obtained from colon cancer patients.** (**a**) Typical 2-DE images of whole proteomes extracted from two different tissues. The protein identified by the white arrow in the enlarged image was overexpressed in colon tumor tissues. MALDI-MS analysis of the protein highlighted in the upper panel unambiguously identified it as caldesmon. (**b**) Differential expression of the 65 kDa isoform of caldesmon (*l*-CaD). Two caldesmon isoforms, *h*-CaD (80 kDa) and *l*-CaD (65 kDa) were dominantly detected in tissues from colon cancer patients (CPs) by western blot analysis. The level of the *l*-CaD was significantly increased in colon tumors (T) compared with normal colon mucosa (N).

Whole-tissue protein extracts from colon mucosa samples and colon tumors were analyzed by 2-DE (Figure 1a). A protein spot with a molecular weight of 100 kDa and a p*I* of 7.0 was expressed at a lower level in colon mucosa tissues than in colon tumors (Figure 1a). The spot was excised from the gel, digested with trypsin, and analyzed by MALDI-MS to determine the peptide mass. A Swiss-Prot database search identified the protein as caldesmon (CaD) (Figure 1a, Additional file 1: Figure S1). Up-regulation of caldesmon in colon tumor tissues was confirmed by Western blot analysis (Figure 1b). The caldesmon protein level was higher in colorectal cancer tissues compared with the corresponding normal colon mucosa samples in six colorectal cancer patients. Notably, the level of the 65 kDa isoform of caldesmon (*l*-CaD) was significantly higher in colon tumor tissue than in normal colon mucosa (Figure 1b).

### Correlation between increased *l*-CaD levels in colorectal cancers and in liver metastases

**Figure 2:**
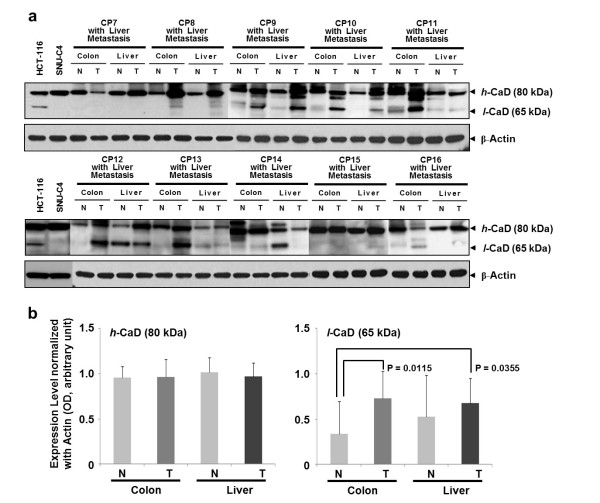
**Aberrant expression of *l*-CaD in primary tumors and liver metastases.** (**a**) Expression pattern of *l*-CaD (65 kDa) among normal colon mucosa, colon tumors, normal liver, and liver metastases. (**b**) Up-regulation of *l*-CaD in primary colon tumors and liver metastases. The two isoforms were detected on western blots, and their relative expression levels were determined by normalization to actin. Although expression of *h*-CaD (80 kDa) isoform did not differ among the sample groups, expression of *l*-CaD (65 kDa) was significantly increased in colon tumors (*P* = 0.0115) and liver metastases (*P* = 0.0355) compared with normal colon mucosa samples.

Western blot analysis of colorectal cancers and the corresponding hepatic metastases revealed higher expression of *l*-CaD (65 kDa) in the cancer tissues than in normal colon mucosa (Figure 2a). Relative expression levels of CaD in normal colorectal mucosa, colorectal cancers, normal liver, and the corresponding liver metastases (*n* = 10 per group) were determined by normalization to actin expression. For *h*-CaD (80 kDa), the mean relative level showed no inter-group difference. In contrast, the mean *l*-CaD level were significantly higher in both colorectal cancers (*P* = 0.0115) and liver metastases (*P* = 0.0355) compared with the levels in normal tissues (Figure 2b).

### Differential expression of *l*-CaD according to tumor regression grade

**Figure 3:**
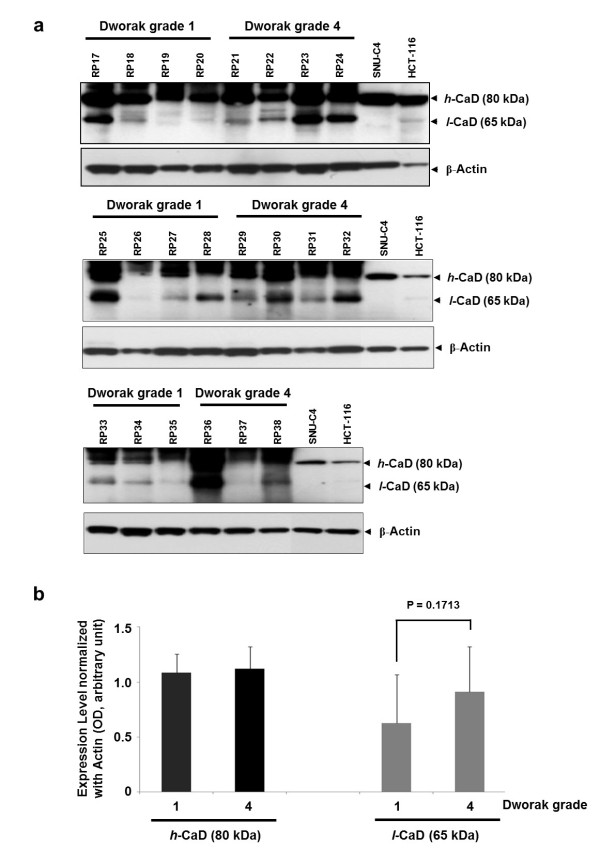
**Expression of *l*-CaD in rectal cancer patients according to chemoradiation response.** (**a**) Expression pattern of *l*-CaD (65 kDa) in tissues obtained from rectal cancer patients (RPs), according to Dworak tumor regression grade. Human HCT-116 and SNU-C4 colon cancer cells were used as positive controls for the two caldesmon isoforms. (**b**) Differential expression of *l*-CaD (65 kDa) between Dworak tumor regression grade 1 and 4 tumors. The two caldesmon isoforms were detected on Western blots, and their relative expression levels were determined by normalization to actin. Expression of *h*-CaD (80 kDa) did not differ according to regression grade, whereas expression of *l*-CaD (65 kDa) was slightly increased in Dworak tumor regression grade 4 tumors but did not reach statistical significance.

The response to chemoradiotherapy was evaluated based on the tumor regression grade, where grade 1 indicates a poor response, and grade 4 is a complete response. In Western blot analyses of 22 rectal cancer tissue samples, *l*-CaD showed differential expression between tumors with a poor response and those with a complete response (Figure 3a). The expression levels of caldesmon in the 22 rectal cancer tissues were normalized to actin expression. The mean relative level of *h*-CaD expression did not differ between groups. Although *l*-CaD expression tended to be higher in tumors with regression grade 4 compared with grade 1 tumors, the difference was not statistically significant (*P* = 0.1713; Figure 3b).

### Increased expression of *l*-CaD in human colon cancer cell lines linked to 5-FU and radiation susceptibility

**Figure 4:**
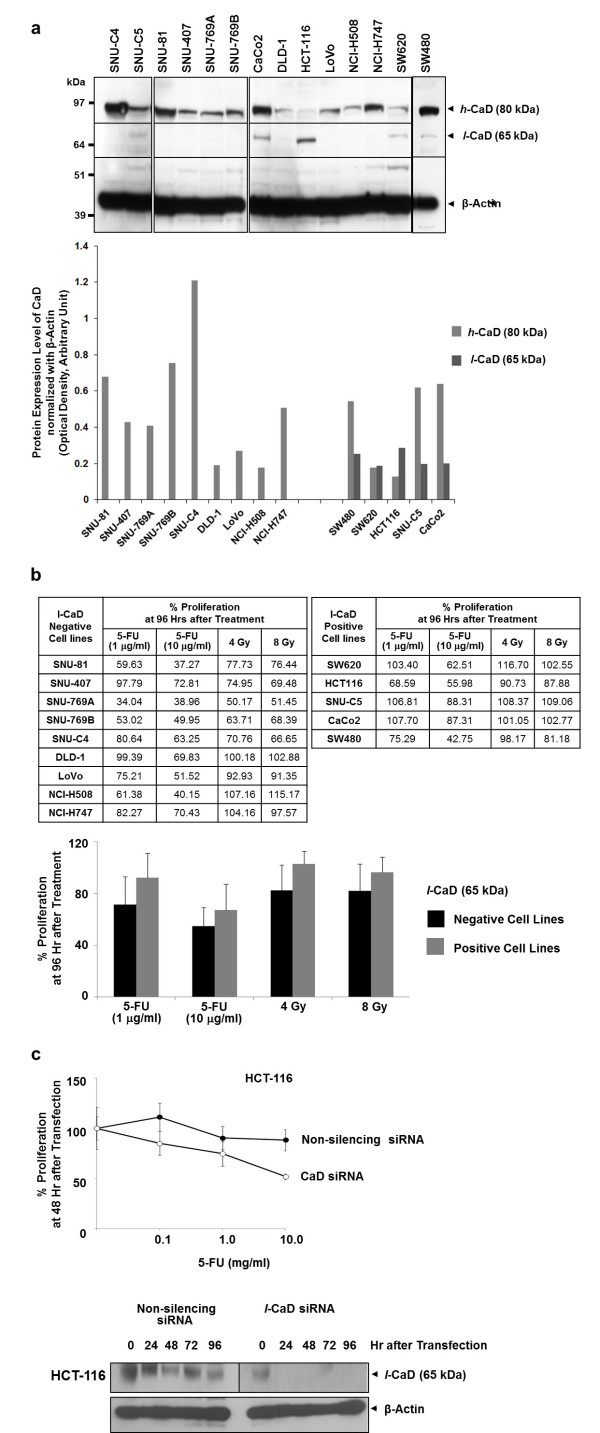
**Expressional relevance of *l*-CaD to 5-FU and radiation response in human colon cancer cell lines.** (**a**) Expression of caldesmon isoforms in 14 human colon cancer cell lines. Unlike in colon cancer tumor, most cell lines tested did not express *l*-CaD (65 kDa). Moderated immunoreactive signal of *l*-CaD (65 kDa) was detected only in SNU-C5, CaCo2, HCT-116, SW480 and SW620. (**b**) Relative poor response of *l*-CaD (65 kDa) positive cell lines to 5-FU and radiation. Expressional levels of *h*-CaD (80 kDa) isoforms were correlated neither with 5-FU, radiation nor invasion (data not shown). However, cell lines expressed *l*-CaD (65 kDa) (SNU-C5, CaCo2, HCT-116, SW480, SW620) showed relatively poor response to 5-FU and radiation compared *l*-CaD (65 kDa) negative cell lines (SNU-C4, SNU-81, SNU-407, SNU-769A, SNU-769B, DLD-1, LoVo, NCI-H508, NCI-H747). (c) Effect of *l*-CaD (65 kDa) suppression on 5-FU. Treatment of 5-FU treatment after *l*-CaD siRNA transfection increased 5-FU susceptibility in HCT-116. However, such artificial suppression of *l*-CaD (65 kDa) did not alter the response to radiation in HCT-116 (data not shown).

Expression level of CaD was also investigated in in thirteen human colon cancer cell lines. Depending on a cell type variable expression pattern of *h*-CaD was found (Figure 4a). However, unlike in colon cancer tumor, *l*-CaD was not detected in most cell lines, and only four cell lines including SNU-C5, CaCo2, HCT-116, SW480 and SW620 showed moderated expression of *l*-CaD (Figure 4a). When cell lines were divided two groups, *l*-CaD negative (SNU-C4, SNU-81, SNU-407, SNU-769A, SNU-769B, DLD-1, LoVo, NCI-H508, NCI-H747) and positive (SNU-C5, CaCo2, HCT-116 and SW620) cell lines, relative poor response to 5-FU and radiation was monitored in *l*-CaD positive cell lines (Figure 4b). However, those differences were not statistically significant, and expressional level of *h*-CaD was also not correlated either with 5-FU, radiation or invasion (data not shown). To clarify whether *l*-CaD plays a role for malignancy, the invasiveness and 5-FU or radiation response of HCT-116 colon cancer cell line with higher expression of *l*-CaD was investigated at 48 hr after artificial suppression of *l*-CaD by siRNA transfection. 5-FU treatment after *l*-CaD suppression increased 5-FU susceptibility in HCT-116 (Figure 4c), but such artificial suppression of *l*-CaD did not change the invasiveness and response to radiation in HCT-116 (data not shown).

### Effect of artificial suppression of *l*-CaD on the expression of main cellular signaling molecules

**Figure 5:**
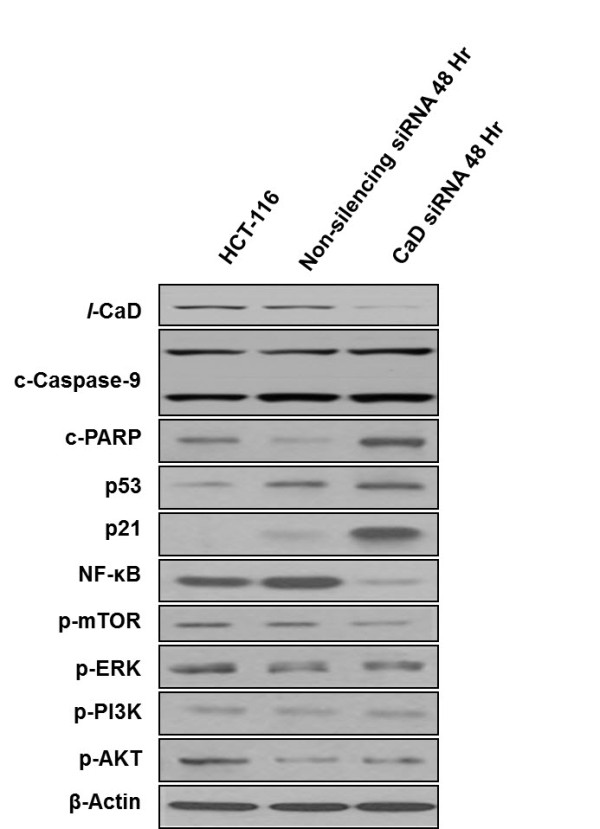
**Effect of *l*-CaD silencing on expression of main cellular signaling proteins.** HCT-116 cells was transfeced by CaD siRNA, and whole proteins was extracted at 48 hr after transfection to investigate the expressional change of nine cellular signaling molecules. The expression levels of c-PARP and p21 were increased after *l*-CaD silencing, but NF-kB and p-mTOR were decreased compared to the non-silencing siRNA transfected cells.

Expression level of 9 major cell signaling molecules was investigated at 48 hr after artificial suppression of *l*-CaD by siRNA transfection. The *l*-CaD siRNA transfected cells showed significantly higher expression levels of c-PARP and p21 than the non-silencing siRNA transfected cells; however, NF-kB and p-mTOR were decreased by the transfection (Figure 5).

## Discussion

Our recent 2-DE-based proteome approach showed that caldesmon was expressed at higher levels in colon tumor tissues than in normal colon mucosa samples (Figure 1). Western blot analysis revealed that two isoforms of CaD, *h*-CaD (80 kDa) and *l*-CaD (65 kDa) were dominantly expressed in colon cancer tissues (Figure 1b). However, only *l*-CaD was significantly higher in both colorectal cancers (*P* = 0.0115) and liver metastases (*P* = 0.0355) than in the corresponding normal mucosa samples (Figure 2). Alternative splicing is a key mechanism for creating complex proteomes from a relatively limited number of genes. It has been estimated that about three-quarters of all human genes undergo alternative splicing [20-22], which may affect the function, localization, binding properties, and stability of the expressed proteins [23]. Alternative splicing can also lead to transcript degradation, thereby abrogating protein expression. For certain serine/arginine-rich protein splicing factors, inclusion of a particular exon causes mRNA degradation by nonsense-mediated decay [24,25]. Tumor-specific CaD splice variants have been reported in tissues from patients with colon, urinary bladder, and prostate cancers [26]. *In silico* protein predictions have suggested that tumor-specific splice variants encode proteins with potentially altered functions, indicating that they may be involved in pathogenesis and hence represent novel therapeutic targets [26]. Among the CaD isoforms, about 67 kDa isoform of *l*-CaD is a major calmodulin-binding protein present throughout the normal gastrointestinal tract and in neoplastic human tissues [27]. Calmodulin is a ubiquitous cytoplasmic protein that mediates many actions of calcium in intestinal tissues, including the regulation of growth and differentiation of normal and neoplastic cells [27]. Significantly suppressed expression of *h*-CaD and the actin-binding protein calponin h1 has been reported in blood vessels of malignant melanomas [28]. In malignant melanoma patients, the expression of *h*-CaD was inversely correlated with the frequency of metastasis and positively correlated with the survival rate [28]. The suppression of *h*-CaD expression in the blood vessels in malignant melanoma implies structural fragility of the vessels, which could result in their easy penetration by tumor cells. Defective expression of *h*-CaD was therefore suggested as a marker for metastatic potential and poor prognosis in melanoma [28]. Our present results cannot clearly assign the role(s) of individual CaD iosforms in colon cancer, but suggest that differential expression of isoforms may be one of the causes leading to tumor characteristics.

Interestingly, differential expression of *l*-CaD was also monitored in the tissues from preoperative rectal cancer patients (Figure 3). Higher expression of *l*-CaD was found in tumors of regression grade 4, which indicates a good chemotherapy response, than in regression grade 1 tumors, but the difference was not significant (*P* = 0.1713) (Figure 3). Recent studies have shown that higher gene expression of CaD, methylenetetrahydrofolate reductase, and multidrug-resistance protein 1 was associated with a response to chemotherapy in esophageal carcinoma [29,30]. Furthermore, our results showing the change of 5-FU response in colon cancer cells by artificial suppression of *l*-CaD strongly supports that *l*-CaD may play a role for chemotherapy response (Figure 4c).

The phosphorylation of CaD by p34^cdc2^ kinase results in dissociation of CaD from actin filaments and possibly plays an important role in disassembly of actin cytoskeleton during mitosis [31]. Therefore, the dysregulation of *l*-CaD may lead to the change of proliferative characteristics in cancer cells in response to radiation or anti-cancer drug treatment. The *l*-CaD suppression in HCT-116 cells caused up-regulation of c-PARP and p21 compared to the non-suppressed cells (Figure 5). p21 as a CDK inhibitor 1 regulates cell cycle by inhibiting cyclin-CDK1 or 2 complexes [32], and also can induce cellular growth arrest or apoptosis [33,34]. NF-KB is a “rapid-reacting” primary transcription factor, and mTOR is also well-known protein kinase involved in cell growth and proliferation [35,36]. Therefore, our *l*-CaD suppressed HCT-116 cells showed characteristics similar to the cells under the apoptotic process. The *l*-CaD siRNA transfected cells also showed relatively high level of c-PARP, which is involved in DNA repair [37]. However, if too much PARP is activated, PARP can deplete cellular NAD + and induce necrotic cell death [37]. Thus, an increased level of c-PARP after *l*-CaD suppression may represent the necrotic cell death as well.

## Conclusions

Our overall data strongly support the positive link between up-regulated expression of *l*-CaD and increased malignancy of colorectal cancer. Dysregulated expression of *l*-CaD may induce metastatic properties and change CRT susceptibility in colorectal cancer cells. The expression level of *l*-CaD may also be helpful in predicting the response of upper gastrointestinal carcinomas to neoadjuvant chemotherapy. However, the molecular mechanism by which it modulates a chemotherapy response has to be further verified.

## Competing interests

The authors declare that they have no competing interests.

## Authors’ contributions

DYK and BCY participated in the design of the study. KHK, SGY and WKK performed research. All authors provided study material and were involved in manuscript writing; they read and approved the final manuscript. KHK and BCY drafted the manuscript.

## Pre-publication history

The pre-publication history for this paper can be accessed here:

http://www.biomedcentral.com/1471-2407/12/601/prepub

## Supplementary Material

Additional file 1 **Figure S1.** Identification of proteins indicated in Figure 1a by MALDI-TOF analysis. Click here for file
