# Supplementary figures and images for: Up-regulated expression of l-caldesmon associated with malignancy of colorectal cancer
Source: BMC Cancer. 2012 Dec 17;12:601. doi: 10.1186/1471-2407-12-601 (PMC3572427; doi:10.1186/1471-2407-12-601)

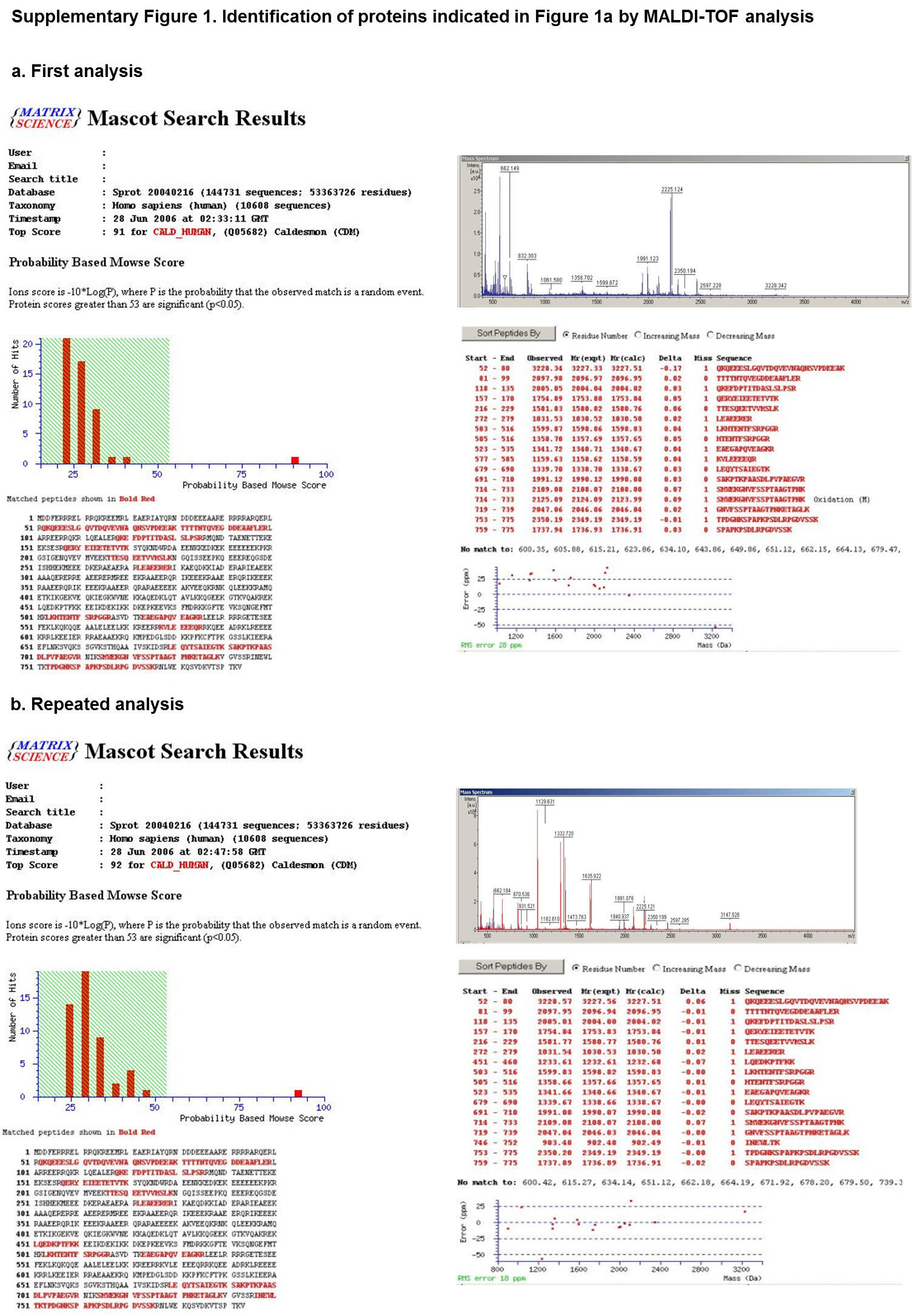

Supplement: Additional file 1 — Figure S1. Identification of proteins indicated in Figure 1a by MALDI-TOF analysis. [file 1471-2407-12-601-S1.jpeg]
